# Lost in .*VCF Translation. From Data Fragmentation to Precision Genomics: Technical, Ethical, and Interpretive Challenges in the Post-Sequencing Era

**DOI:** 10.3390/jpm15080390

**Published:** 2025-08-20

**Authors:** Massimiliano Chetta, Marina Tarsitano, Nenad Bukvic, Laura Fontana, Monica Rosa Miozzo

**Affiliations:** 1 A.O.R.N. A. Cardarelli Hospital’s Laboratory of Medical Genetics and Genomics, 80131 Naples, Italy; marina.tarsitano@aocardarelli.it; 2 U.O.C Genetica Medica, Azienda Ospedaliero Universitaria Consorziale Policlinico di Bari, 70124 Bari, Italy; nenadbukvic@virgilio.it; 3 Medical Genetics Unit, ASST Santi Paolo e Carlo, 20153 Milan, Italy; laura.fontana@unimi.it; 4 Medical Genetics, Department of Health Sciences, Università degli Studi di Milano, 20122 Milan, Italy; monica.miozzo@unimi.it

**Keywords:** genomic medicine, Variants of Uncertain Significance (VUS), ethical challenges

## Abstract

**Background:** The genomic era has transformed not only the tools of medicine but the very logic by which we understand health and disease. Whole Exome Sequencing (WES), Clinical Exome Sequencing (CES), and Whole Genome Sequencing (WGS) have catalyzed a shift from Mendelian simplicity to polygenic complexity, from genetic determinism to probabilistic interpretation. This epistemological evolution calls into question long-standing notions of causality, certainty, and identity in clinical genomics. Yet, as the promise of precision medicine grows, so too do the tensions it generates: fragmented data, interpretative opacity, and the ethical puzzles of Variants of Uncertain Significance (VUSs) and unsolicited secondary findings. **Results:** Despite technological refinement, the diagnostic yield of Next-Generation Sequencing (NGS) remains inconsistent, hindered by the inherent intricacy of gene–environment interactions and constrained by rigid classificatory systems like OMIM and HPO. VUSs (neither definitively benign nor pathogenic) occupy a liminal space that resists closure, burdening both patients and clinicians with uncertainty. Meanwhile, secondary findings, though potentially life-altering, challenge the boundaries of consent, privacy, and responsibility. In both adult and pediatric contexts, genomic knowledge reshapes notions of autonomy, risk, and even personhood. **Conclusions:** Genomic medicine has to develop into a flexible, morally sensitive paradigm that neither celebrates certainty nor ignores ambiguity. Open infrastructures, dynamic variant reclassification, and a renewed focus on interdisciplinary and humanistic approaches are essential. Only by embracing the uncertainty intrinsic to our biology can precision medicine fulfill its promise, not as a deterministic science, but as a nuanced dialogue between genes, environments, and lived experience.

## 1. Introduction

Whole Exome Sequencing (WES), Clinical Exome Sequencing (CES), and Whole Genome Sequencing (WGS) have drastically changed the technological approach to disease understanding, thereby transforming the epistemological basis of clinical practice [1]. Today, clinical genomics is poised to adopt a more complex, multigenic comprehension of disease that transcends the mere observation of symptoms and the investigation of single-gene variants [2]. While this paradigm shift has enhanced both diagnostic accuracy and therapeutic stratification, it has also introduced substantial conceptual and technical challenges. Although high-throughput sequencing has made genomics analysis more accurate, turning raw data into clinical actionable knowledge remains demanding. Each dataset must be filtered, standardized, and cross-validated, through different stages from sequencing to therapeutic application. However, no analytical step occurs in isolation: laboratory protocols, data generation, bioinformatic algorithms, and specific expertise collectively interact, ultimately determining the outcome and the quality of the result [3].

A central innovation of this article lies in critically examining how variant interpretation operates at the intersection of evolving genomic technologies, variable informatic frameworks, and heterogeneous clinical settings. The conceptual conflicts between clinical ambiguity and data-driven precision were specifically highlighted when developing methods to combine machine-readable genomes with the complex reality of medical decision making.

Beyond interpretative challenges, a significant barrier remains the fragmentation of genomic data. The lack of interoperable platforms and shared databases limits the cross-study variant comparison and classification, delaying both potential discoveries and subsequent advancements in diagnosis and therapy. A systematic review of research on variant reclassification and patient recontact in medical genetics from 2013 to 2023 illustrates the dynamic nature of genetic interpretation. It found that up to 40% of clinically reported variants are reclassified within five years [4]. Furthermore, a 2023 study involving 500 cardiology patients showed that clinical care changed in 12% of cases when Variants of Uncertain Significance (VUSs) in the MYH7 gene were reclassified as pathogenic following functional testing [5].

Despite these advancements, the promise of precision medicine is somewhat hindered by the fact that genomic data is still primarily isolated within proprietary platforms that are incompatible and provide restricted accessibility [6,7]. To fully harness this potential demand, genomic medicine must integrate new findings, technological advancements, and collective expertise with a framework that acknowledges the ever-evolving nature of genetic knowledge. Yet the creation of open and interoperable infrastructures, and the collaborative efforts they presuppose, remains largely theoretical, retarding progress in risk assessment, as well as in the reclassification and clinical interpretation of genomic variants [8].

To overcome data fragmentation and maximize the benefits of sequencing technologies, clinical decision making must shift toward a highly adaptive framework that continuously incorporates emerging genomic data while responsibly navigating the associated ethical complexities. Preserving ethical primacy in this evolution demands a careful balance between informed consent, patient rights, and the responsible management of genetically ambiguous findings [9,10].

Despite the advances of Next-Generation Sequencing (NGS), its diagnostic yield remains variable, ranging from 25% to 50%. These constraints present a significant cognitive challenge, arising from intrinsic technical limitations of the method, allelic heterogeneity, and the complex interplay of multiple contributors to disease presentation [11]. These constraints present a significant cognitive and interpretative challenge, arising from intrinsic technical limitations such as incomplete coverage, GC-rich or repetitive regions, and short-read sequencing’s inability to detect complex structural variants or low-level mosaicism. Additional obstacles include allelic heterogeneity, limited phenotypic specificity, and the presence of VUSs, which can account for 30–40% of findings in clinical exome sequencing, delaying actionable diagnosis. Moreover, the yield is notably lower in non-European populations, largely due to the underrepresentation of diverse ancestries in reference databases, further amplifying inequities in genomic medicine [12,13].

Bioinformatics tools that integrate curated databases, such as Online Mendelian Inheritance in Man (OMIM) and Human Phenotype Ontology (HPO), serve as indispensable mediators of genomic knowledge. By converting large-scale sequencing data into structured, analyzable datasets, they enhance diagnostic precision. However, their accuracy on established classification schemas introduces an intrinsic limitation: a pronounced focus on already classified variants can obscure novel or insufficiently characterized genetic determinants [14].

This disjunction between the analogue ambiguity of clinical observations and the digital precision of clinical classification (or taxonomic ontologies?) points to a deeper issue in medicine itself. Whereas clinical diagnosis often resides in a fluid, context-dependent space, terminological systems such as HPO and OMIM impose rigid machine-readable boundaries on disease entities. This discrepancy raises two critical considerations: first, the extent to which prevailing informatics frameworks are reshaping, perhaps constraining, our conception of disease; and second, the possibility that sizeable portions of the genomic landscape remain invisible precisely because they fall outside the detection limits of these structured paradigms [15,16].

A methodological framework that embraces, rather than resists, ambiguity is crucial if the aim of genomic medicine is to achieve both accurate diagnosis and the ongoing refinement of disease paradigms. The shift from monogenic to polygenic perspectives constitutes more than a scientific advancement; it marks a profound conceptual transformation. By undermining classical genetic determinism, it replaces reductionist views with a framework that foregrounds the intrinsic biological complexity [17].

The classical Mendelian framework, once valued for its clarity and simplicity, now shows clear limitations. Although still useful in specific contexts, it cannot fully capture the vast spectrum of human health and disease [14]. In this emerging model, genes act not as isolated determinants of fate but as dynamic components within an intricate network of molecular and environmental interactions [18].

Polygenic Risk Scores (PRSs) exemplify this conceptual shift: rather than delivering absolute diagnostic likelihoods, they yield probabilistic estimates shaped by a wide range of genetic polymorphisms and environmental factors.

Genetic predisposition now represents a spectrum of probabilities rather than a predetermined fate. The genome, once considered a fixed script at conception, now resembles a dynamic score, constantly revised by external factors such as diet, stress, social environment, and chance. In this perspective, the “genetic self” is a dynamic interplay between inherited patterns and lived experience, rather defined by a static blueprint [19].

The concept of health shifts from a binary condition of wellness or disease to a dynamic spectrum influenced by factors beyond personal control [20]. This new concept raises critical existential and ethical questions. Should our concept of personal responsibility be recalibrating in the face of genetic predisposition? How can we balance the potential psychological burden of knowing one’s hereditary risks with the right to be informed? Crucially, we move from monogenic to multigenic and multifactorial frameworks representing not only a technical advance but also an acknowledgment of the intrinsic complexity of human genetics.

## 2. Genetic Uncertainty: Balancing Knowledge, Ethics, and Autonomy in the Era of VUSs and Secondary Findings

The management of VUSs represents the epistemic complexity of contemporary genomics, confronting both patients and clinicians with the limit of current knowledge. VUSs can be currently perceived as ambiguous spaces in genomic knowledge; ambiguous variants that evade definitive classification and persist in an epistemologically indeterminate state [21].

VUSs pose a non-trivial dilemma for therapeutic decision making, compelling clinicians to balance uncertain genomic data against the need for concrete clinical action. In the absence of a definitive interpretation, clinicians must reconcile between evidence and conjecture, an ambivalence that may lead to anxiety for both patients and professionals, as crucial health decisions remain suspended in uncertainty [22].

The ethical and psychological dimensions of VUSs extend beyond immediate therapeutic choices. Patients must confront the unsettling realization that their genetic history is incomplete, painted with possibilities that may never materialize into disease or may remain indefinitely ambiguous. This uncertainty can provoke excessive anxiety and, in some cases, drive individuals toward unwarranted medical interventions. Accordingly, the ethical challenge for healthcare providers is reduction of suffering while conveying genetic information responsibly, striking a balance that recognizes uncertainty without amplifying it [23].

The need to reclassify VUSs highlights the dynamic nature of genetic science. Although such work demands considerable time, funding, and cross-collaboration between scientific communities, three approaches remain indispensable: family segregation analysis, functional studies including in silico modeling, and large-scale population studies. Despite the rate at which new information emerges, it lags behind the urgency of clinical needs, leaving uncertainty for both clinicians and patients [24].

The evolving nature of genomic knowledge is evident in the periodic re-evaluation of VUSs: a variant that remains uncertain today may be reclassified as pathogenic or benign tomorrow. Given this fluidity, the validity of medical diagnoses and the mechanisms for communicating these reclassifications must be continuously reassessed [25]. To ensure that patients remain both informed and supported as their genomic profiles are reinterpreted, laboratories should develop adaptable protocols that acknowledge the transient nature of many genetic insights and, when appropriate, consider the generation of dynamic reports. Moreover, geneticists should explain during the initial informed consent process the possible uncertainty of the genetic test.

Real-world clinical examples demonstrate how decisions can still be made based on a VUS. For instance, in a case reported by Shirts et al. (2016), a VUS in the *TP53* gene was identified in a patient with early-onset breast cancer; despite its uncertain classification, increased surveillance was initiated due to strong family history and clinical suspicion [26]. In another study, Saitoh et al. (2020) described a pediatric patient with epileptic encephalopathy and a VUS in *SCN1A* and *SCN2A* genes. Functional studies and phenotypic concordance led to a change in therapy that significantly improved seizure control [27]. These cases underscore the importance of integrative interpretation and clinical judgement when managing VUSs.

VUSs and, more broadly, indeterminate genomic findings pose ethical challenges in clinical reporting, as they intersect with knowledge, ambiguity, and professional judgment. This is more evident when NGS is applied to both adult and pediatric populations, because genetic information has the power to alter identity, influence decision making, and redefine fundamental aspects of personhood.

In pediatrics, where the patient is unable to provide informed consent, genetic testing acquires profound ethical implications. Parents and guardians, entrusted with decision making, act as intermediaries between medical expertise and the child’s future autonomy. A pivotal question arises: how much genomic information should be disclosed to an individual who lacks the cognitive maturity to process it? This dilemma highlights the tension between the potential benefits of genomic knowledge and the ethical principle of the right not to know, which protects individuals from the psychological burden of ambiguous or distressing results [28]. In adults, by contrast, the ethical center of gravity shifts toward self-determination.

The consequences of genetic discoveries in children extend beyond questions of ethical autonomy. They influence future reproductive choices, psychological development, and societal perceptions of health across the lifetime. The risk of genetic determinism is a significant concern: does awareness of a genetic predisposition shape an individual’s identity and self-perception, or does it merely offer an opportunity for proactive intervention? Carrier status illustrates the point. Largely inconsequential in childhood, it takes on critical importance in adulthood, informing family planning decisions and reshaping intergenerational relationships [28]. For adults undergoing genetic testing, the focus shifts toward self-determination. Individuals may choose either to pursue or to avoid genetic insights. Unlike pediatric cases, where information is passively received, adult testing involves a more deliberate engagement with one’s biological heritage. However, this autonomy comes with consequences: information that can empower clinical vigilance may also provoke anxiety, disrupt life plans, and influence decisions about reproduction and healthcare [29].

To ensure that results are effectively brought to the patient’s bedside, a structured reporting workflow must be implemented, integrating laboratory pipelines, interdisciplinary dialogue, and personalized counseling. This could include a clinical integration of genomic data following a structured pathway that begins with variant identification from sequencing data (e.g., *.vcf), followed by classification according to specific guidelines and evaluation through multidisciplinary discussion involving bioinformaticians, geneticists, and clinicians to contextualize findings within the patient’s phenotype. The resulting interpretation is formalized in a clinical report, which is subsequently communicated to the patient through genetic counseling, ensuring that the uncertainty surrounding a VUS is clearly explained and appropriately managed. A robust reporting flow enables real-time communication between laboratories and clinical teams, ensuring that uncertain results like VUSs are contextualized within the patient’s phenotype.

Moreover, the principle of free and informed decision making is complicated by the social and economic ramifications of genomic information. In some legal and cultural contexts, genetic test results extend beyond personal health, affecting insurance eligibility and employment prospects, thereby raising the specter of genetic discrimination. Should an individual’s genomic profile legitimately affect insurability or employability? [29].

The impact of genetic studies on families presents one of the most intricate moral dilemmas. Identification of a single pathogenic variant in one individual has effects throughout the biological lineage, directly implicating relatives who may be unaware of their own risk. Ethical obligations to inform high-risk relatives introduce questions of privacy, responsibility, and the interplay between personal autonomy and familial duty. Where, then, does personal liberty yield to familial responsibility? Should individuals have full control over their genetic information, or does an ethical obligation exist to disclose clinically relevant findings to at-risk relatives who could benefit from that knowledge? [30].

In dominant genetic diseases, identification of an inherited variant can reinforce a sense of biological inevitability: individuals confront the genetic legacy of previous generations and the ethical dilemma of possibly transmitting that legacy to their offspring. Beyond the ethical dilemma of potentially passing on such variants, affected individuals must also grapple with the psychological burden of genetic inheritance. By contrast, de novo variants introduce unpredictability and a perception of genetic singularity. This discontinuity can be perceived either as a form of biological solitude or as a sense of liberation, as the absence of inherited risk also means no transmission of disease or legacy to potential progeny [30].

Recessive disorders, on the other hand, expose the latent complexity of inheritance. Healthy carriers, often unaware of their genetic status, unknowingly possess the potential for disease expression in future generations. Additionally, maternal and sex-linked inheritance introduce further asymmetries, that shape distinct risks profiles, adding layers of ethical and clinical complexity to counseling practice [30].

Beyond personal implications, transmission risk plays a crucial role in genetic counseling, shaping the reproductive choices of individuals seeking to understand their family’s genetic future. In this intricate mosaic, genetics function both as a life science and as a narrative of human destiny, mediating between determinism and variability.

“Second hit” findings present a further challenge. These unexpected variants, uncovered incidentally through molecular diagnostics, occupy the space between knowledge and the right to not to know. They may expand medical possibilities while simultaneously raising profound existential questions: should one resist crossing this threshold of knowledge or embrace the hidden information encoded in one’s genome? In transcending its utility such discoveries expose the tension between the desire to know and the right to remain unaware [31].

The management of secondary findings raises fundamental issues related to personal autonomy. Genomic testing can reveal predispositions to a wide range of conditions, including cancer, cardiovascular diseases, and adverse drug reactions. However, genetic knowledge does not always translate into immediate clinical benefit or into clear opportunities for prevention or treatment. Some genetic information can have a significant psychological impact, especially when it concerns conditions for which no effective therapy currently exists. An example was the discovery of mutations in the BRCA1 and BRCA2 genes which entail a substantial increase in the risk of developing cancers, mainly breast and ovarian neoplasms, often leading to drastic preventive decisions such as prophylactic mastectomy and/or oophorectomy. Other situations, such as genetic predisposition to inherited cardiovascular conditions (e.g., long QT syndrome, Brugada syndrome, or hypertrophic cardiomyopathy), can result in a persistent state of anxiety, even in the absence of clinical symptoms. In such cases, the disclosure of a genetic risk can be experienced as a “Sword of Damocles” hanging over the individual’s future.

Not every genetic variant provides clear or useful information; many remain probabilistic and lack direct clinical relevance. This uncertainty raises critical questions about the disclosure of genetic data: should findings be communicated only when they confer a clear therapeutic or preventive benefit, or should transparency prevail in anticipation of future significance? [31].

Similar to VUSs, incidentally, detected second hits carry profound social and psychological implications. Awareness of a latent genetic predisposition can induce genetic anticipation, leading to both existential anxiety and proactive health behavior. Because genomic information is intrinsically familial, and rarely isolated to an individual, disclosure inevitably impacts family dynamics, raising concerns about shared genetic risk, privacy, and responsibility. Does the duty to inform relatives override an individual’s right to genomic autonomy? [32].

## 3. Genetic Roulette: Global Discrepancies in Variant Interpretation and Their Impact on Medicine and Law

Despite sustained efforts by leading scientific organizations like the American College of Medical Genetics and Genomics (ACMG) collaborating with the Association for Molecular Pathology (AMP) and the European Society of Human Genetics (ESHG), no overarching consensus yet governs the clinical interpretation of sequence variants [33]. Although each organization has outlined classification criteria, their approach to clinical contextualization diverges, revealing a persistent tension between the need for rigorous systematization and the need for flexible, context-sensitive interpretation [34] (Table 1).

The ACMG framework privileges a tightly structured, evidence-weighted model, whereas the ESHG adopts a more fluid strategy, emphasizing clinical relevance and the use of European databases, such as LOVD (https://www.lovd.nl/, accessed on 20 March 2025) and UMD (http://www.umd.be/lsdb.html, accessed on 20 March 2025), particularly in the management of VUSs within European cohorts [35] (Table 2).

Moreover, both schemes expand the interpretive spectrum by incorporating intermediate categories such as “likely pathogenic” and “likely benign”, thereby widening, rather than narrowing, the zone of ambiguity surrounding variant classification [36].

Reliance on open repositories, above all ClinVar, the community’s most widely consulted database, further compounds this divergence. Although ClinVar is a vital tool for gathering genetic data, it has limitations due to heterogeneous data sources and variable interpretive standards [37]. The star-rating system, intended to convey consensus and evidentiary strength, paradoxically introduces an additional element of uncertainty [38], amplifying discrepancies both in the interpretation level (conflict of interpretation) and in pathogenicity classification (conflict of classification of pathogenicity) [39]. Consequently, data reliability is diminished, and the burden of converting ambiguous annotations into clinically applicable insights rests solely with the clinician [40].

The consequences of this ambiguity extend beyond the clinical domain and impact ethical and legal facets as well [41]. The ACMG discourages the communication of VUSs to patients unless they are relevant in specific contexts or part of clinical studies, whereas the ESHG and SIGU adopt a more permissive approach, allowing VUS disclosure when they may acquire clinical significance over time or if they have a relevant impact. Additionally, SIGU promotes integration with Italian and European genetic databases, emphasizing the value of clinical context and genetic counseling to reduce discrepancies between laboratories [42,43,44].

**Table 1:** ESHG NGS variant-classification system. The ESHG “ABC” classification is a stepwise system applicable to any genetic variant [43]. Classification is first functional (Step A), assessing the predicted biological consequence, and then clinical (Step B), focusing on genotype–phenotype correlation. In Step C (integrated grading), each variant is reassigned to an integrated class (A–F, 0, X) by combining its functional score (Step A) with its clinical score (Step B); the resulting class directly guides reporting decisions.

**a. ESHG NGS Variant Classification—Step A (Functional)**		
**Functional Class**	**Score**	**Operational Definition**
Functional VUS (fVUS)	0	Variant of unknown functional significance
Normal function (NF)	1	High-frequency variant with no reason to suspect recessive/hypomorphic role
Likely normal function (LNF)	2	Moderate-frequency variant with no reason to suspect recessive/hypomorphic role
Hypothetical functional effect (HFE)	3	Rare variant that could affect gene function (bioinformatic/biological hints)
Likely functional effect (LFE)	4	Recessive: hypomorphic variant causing disease only in trans with LoF Dominant: variant with likely LoF or other functional importance
Functional effect (FE)	5	Proven LoF/known GoF or dominant-negative variant
**b. ESHG NGS Variant Classification—Step B (Clinical)**		
**Clinical Class**	**Score**	**Operational Definition**
Clinical VUS (cVUS)	0	Variant of unknown clinical significance
Variant of interest (VOI)	1	Dominant candidate variant or single hypomorphic allele in recessive gene
Risk factor	2	Low-penetrance dominant variant or single pathogenic allele matching phenotype
Pathogenic variant	3	Clearly pathogenic variant
Moderate-penetrance pathogenic	4	Dominant variant with 20–40% penetrance
High-penetrance pathogenic	5	Dominant variant with >40% penetrance
**c. ESHG NGS Variant Classification—Step C (Integrated Grading)**		
**Final Class**	**A + B Combination**	**Reporting Recommendation**
0	F0–2	Not reported
F	F3 + C0	Not reported if the gene is unrelated to phenotype
E	F3 + C1/C2 · F4 + C0/C1 · F5 + C0	Variant of interest (optional reporting)
D	F3 + C3 · F4 + C2/C3 · F5 + C1/C2	Low-penetrance/good candidate—report
C	F4 + C4 · F5 + C3	Disease-associated—report
B	F4 + C5 · F5 + C4	Disease-associated, moderate penetrance—report
A	F5 + C5	Disease-associated, high penetrance—report
X	Any F3–5 with C2–5	Secondary/incidental finding

**Table 2:** ACMG/AMP NGS variant-classification system. The ACMG/AMP guidelines classify sequence variants into five tiers (benign→pathogenic) by integrating evidence from population data, computational and predictive algorithms, functional assays, segregation studies, and allelic information [45].

ACMG/AMP Class	Description
Pathogenic (5)	Variant known to cause the disease
Likely pathogenic (4)	Variant very likely to cause the disease, small residual uncertainty
VUS (3)	Pathogenicity uncertain—more data required
Likely benign (2)	Variant very unlikely to cause the disease
Benign (1)	Variant known not to cause the disease

The classification of a variant, even if formally correct according to specific criteria, can lead to inappropriate clinical decisions, for example, unnecessary prophylactic surgeries based on misinterpreted risk or failure to monitor a patient who actually carries a pathogenic mutation, resulting in either overtreatment or missed diagnoses. Such outcomes may carry medico-legal implications, especially given the variability in international regulations regarding genetic data interpretation and disclosure [45]. Discrepancies in genetic interpretation can lead to discordant reports for a patient undergoing testing in different countries, with legal consequences on treatment protocols and insurance decisions [46]. Similarly, companies offering Direct-To-Consumer (DTC) genetic tests must navigate divergent regulations, affecting the legal validity of results across jurisdictions [47].

In some European regions, the principle of patient autonomy and the right to genetic information prevails, whereas in the United States, greater emphasis is placed on protecting patients from the potential psychological impact of uncertain information [45].

This interpretative variability is not limited to Western contexts but extends to global genomic practices. In China, the focus on large-scale genomic studies, such as those conducted by the China National GeneBank (CNGB), which has enabled the identification of population-specific variants, often underrepresented in global databases like ClinVar [48]. In India, the integration of genomic data from a diverse ethnic landscape has highlighted the limitations of classification criteria based on Western standards when applied to genetically distinct populations [49]. In Russia, the development of national genomic databases presents the challenge of harmonizing local variant interpretation with international standards [50]. In Africa, the continent’s remarkable genetic diversity complicates the application of standardized variant classification criteria; consequently, many African variants remain underrepresented in international databases, despite ongoing efforts and initiatives, such as H3Africa, aimed at improving their coverage [51].

In this global context of population-specific variants, the simple dichotomy between US and European is insufficient because allele frequencies and variant classification may vary greatly between populations. Tools such as gnomAD have highlighted how population ancestry affects Minor Allele Frequency (MAF), a crucial parameter for assessing variant pathogenicity [52]. Databases specially designed for a particular population are necessary to prevent the incorrect classification of variations that are rare and potentially pathogenic in one group but benign in another, with obvious clinical consequences. An example of a “founder mutation” with confirmed clinical impact is the *BRCA1* c.68_69delAG (185delAG) variant, which is regarded as harmful and relatively common among Ashkenazi Jews [53]. Other population-specific founder mutations, such as those in the *PALB2* gene among Finnish or French-Canadian individuals, illustrate how ancestry can directly shape genetic risk assessment and management strategies [54].

In these legal ambiguities, universally accepted informed consent is an essential ethical safeguard: it protects clinicians navigating an increasingly complex regulatory landscape while enabling patients to set the boundaries of their genetic knowledge [55].

## 4. The Dark Side of AI in Genomics: Bias, Errors, and the Black-Box Dilemma

The promise of Artificial Intelligence (AI), which includes Machine Learning (ML) and deep learning in genomics, is evident in an era of unparalleled data production [56]. However, its application has significant challenges. The integration of AI (e.g., DeepVariant, AlphaMissense) into variant interpretation pipelines has accelerated analysis but introduced new challenges. Recent benchmarking of AI-driven platforms for variant interpretation has highlighted both their potential and current limitations. In one comparative study of seven tools analyzing 24 clinically validated variants, top performers like SeqOne, CentoCloud, and eVai reached over 90% accuracy, with SeqOne ranking 79% of variants as the top candidate. VarSome Clinical achieved 67% concordance with expert classifications but failed to prioritize 17% of variants, including three CNVs, revealing weak performance in CNV interpretation. Among all tools, Franklin proved most consistent, with 92% of classifications deemed adequate. However, no platform achieved full concordance. CNVs remained particularly challenging, and agreement across platforms occurred for only 4 of 24 variants. These findings echo prior reports, such as VarSome’s 92% ACMG concordance in ideal scenarios, while emphasizing performance gaps for complex variants and underrepresented populations. This underscores the importance of platform-specific validation and regular training on diverse, real-world datasets [57].

Among these are the quality and reliability of the dataset used for modeling training [58,59]. Genomic repositories often contain incomplete or biased information due to underrepresentation of global genetic diversity. Data noise, stemming from sequencing errors, technical inconsistencies across platforms, and contamination, can introduce additional noise [60]. Furthermore, historical databases are riddled with anomalies and duplications, which can distort algorithmic performance, making it difficult to distinguish a truly pathogenic variant from benign ones [61].

Adding to these issues is the intrinsic complexity of the human genome, whose multilayered architecture resists straightforward mathematical abstraction. The interplay among numerous genes and environmental modifiers creates a level of intricacy that current models can capture only in part [62]. As a result, AI systems often identify statistical correlations without truly understanding the underlying biological mechanisms, producing outputs that, although mathematically sound, have limited clinical relevance [63]. This lack of interpretability, the so called “black-box” effect, makes it difficult for geneticists and physicians to place full trust in AI-generated predictions, especially when such predictions are used to make therapeutic decisions [64].

To enhance clinical reliability, AI tools in genomics must be accompanied by transparent performance metrics such as sensitivity and specificity, which are rarely reported in standardized formats [65]. Moreover, poor clinical performance may stem from biased training sets, lack of ethnic representation, or the use of opaque models that limit interpretability, factors that hinder trust and regulatory approval [66].

Ethical and data privacy considerations further complicate the complexity of the deployment of AI in genomics [67]. Genomic information is among the most sensitive categories of personal data, and its incorporation into machine learning pipelines raises serious concerns about security and confidentiality [68]. Additionally, most published genomic studies have predominantly involved individuals of European ancestry, leaving many poorly calibrated for other populations [69]. This bias leads to inaccuracies and, in some cases, produces discriminatory outputs exacerbating existing health disparities [70].

Bridging the gap between statistical performance and clinical applicability will require the development of multimodal models capable of integrating genomic, phenotypic, and clinical data; the implementation of prospective validation studies; and a systematic effort to enhance the representation of ethnically diverse populations within genomic datasets [71]. Within this evolving framework, the burden posed by VUSs is expected to decrease as data repositories grow and interpretive algorithms become more sophisticated. However, in routine clinical practice, VUSs remain frequent findings. Even when clinical evidence or familial segregation analysis supports a likely pathogenic interpretation, such reclassifications are often not communicated to publicly accessible databases such as ClinVar. This lack of data sharing hinders collective advancement in variant interpretation. A coordinated and transparent effort to submit re-evaluated variants would substantially reinforce the genomic knowledge base and improve diagnostic accuracy across populations [72].

## 5. Conclusions

Advancements in sequencing technologies have revolutionized our understanding of genetic disease, yet they have also exposed significant conceptual, ethical, and technical challenges. Progress will depend on interoperable data-sharing frameworks, sustained interdisciplinary collaboration, and a careful balance between the benefits of genomic insights and the rights to privacy and autonomy. Developing global networks for real-time data sharing and variant interpretation, automated re-evaluation procedures (such as quarterly ClinVar updates), and flagging variants with conflicting interpretations to promote greater consistency in clinical classifications and speed up VUS resolution are all examples of future directions. Block-chain-based systems could be employed to share patient data securely and under patient control between organizations.

Practical obstacles extend beyond philosophical debate, highlighting the vulnerability of cross-platform data sharing and analysis. The absence of uniform standardization across sequencing technologies, bioinformatics pipelines, and data-sharing infrastructures fragments the field, impeding the interoperability of genetic insights between institutions. Variants identified on one sequencing platform may not be directly comparable to another owing to differences in computational methods, reference genomes, or laboratory protocols, thereby constraining the reach of precision medicine and forcing costly, duplicative testing that delays diagnosis and exacerbates health inequities.

Interpretation of genomic data remains the critical bottleneck. Assigning clinical significance, especially to VUSs, often stalls therapeutic decision making. Conditions characterized by oligogenic inheritance, somatic mosaicism, or multilayered regulatory mechanisms frequently remain unsolved under a strictly gene-centric model focused on coding regions and protein effects.

This does not mean that such mechanisms fall outside Mendelian logic, many still follow genetic rules, but rather that the phenotype may result from a convergence of multiple factors. Many pathogenic mechanisms exert their effects through gene expression modulation, chromatin organization, or non-coding or regulatory interactions that routine short-read sequencing fails to capture. Even whole genome sequencing, in its current form, often fails to substantially increase diagnostic yield due to the inaccessibility or misinterpretation of certain genomic regions, such as GC-rich or highly repetitive sequences, and due to complex structural variants or low-level mosaicisms. Long-read sequencing technologies and integrative multiomics approaches, including transcriptomics, epigenomics, and 3D chromatin architecture, are therefore required to illuminate these layers.

We are still far from capturing the full scope of genetic contribution to disease. An excess focus on binary VUS reclassification may overlook causal mechanisms residing in unexplored genomic or epigenomic contexts. Apparent “negative findings” may simply reflect an inability to detect RNA editing, chromatin looping, or distant regulatory interactions, the “3D epigenetics” dimension. In this regard, expanding functional validation studies (e.g., using cellular or animal models) remains critical to clarify the biological impact of VUSs and better inform their classification.

Currently, genome sequencing is employed predominantly within research environments; routine, reliable deployment in clinical settings remains aspirational. Realizing the promise of precision medicine will demand not only improved technologies and infrastructures but also a fundamental re-examination of genetic causality and complexity. Equally important will be the implementation of policy frameworks ensuring periodic variant re-evaluation and systematic updates to clinical reports when new evidence becomes available in order to align with evolving knowledge and avoid outdated interpretations in patient care.

## Data Availability

No new data were created or analyzed in this study. Data sharing is not applicable to this article.
